# Emerging concepts in biomarker discovery; The US-Japan workshop on immunological molecular markers in oncology

**DOI:** 10.1186/1479-5876-7-45

**Published:** 2009-06-17

**Authors:** Hideaki Tahara, Marimo Sato, Magdalena Thurin, Ena Wang, Lisa H Butterfield, Mary L Disis, Bernard A Fox, Peter P Lee, Samir N Khleif, Jon M Wigginton, Stefan Ambs, Yasunori Akutsu, Damien Chaussabel, Yuichiro Doki, Oleg Eremin, Wolf Hervé Fridman, Yoshihiko Hirohashi, Kohzoh Imai, James Jacobson, Masahisa Jinushi, Akira Kanamoto, Mohammed Kashani-Sabet, Kazunori Kato, Yutaka Kawakami, John M Kirkwood, Thomas O Kleen, Paul V Lehmann, Lance Liotta, Michael T Lotze, Michele Maio, Anatoli Malyguine, Giuseppe Masucci, Hisahiro Matsubara, Shawmarie Mayrand-Chung, Kiminori Nakamura, Hiroyoshi Nishikawa, A Karolina Palucka, Emanuel F Petricoin, Zoltan Pos, Antoni Ribas, Licia Rivoltini, Noriyuki Sato, Hiroshi Shiku, Craig L Slingluff, Howard Streicher, David F Stroncek, Hiroya Takeuchi, Minoru Toyota, Hisashi Wada, Xifeng Wu, Julia Wulfkuhle, Tomonori Yaguchi, Benjamin Zeskind, Yingdong Zhao, Mai-Britt Zocca, Francesco M Marincola

**Affiliations:** 1 Department of Surgery and Bioengineering, Advanced Clinical Research Center, Institute of Medical Science, The University of Tokyo, Tokyo, Japan; 2 Cancer Diagnosis Program, National Cancer Institute (NCI), National Institutes of Health (NIH), Rockville, Maryland, 20852, USA; 3 Infectious Disease and Immunogenetics Section (IDIS), Department of Transfusion Medicine, Clinical Center and Center for Human Immunology (CHI), NIH, Bethesda, Maryland, 20892, USA; 4 Departments of Medicine, Surgery and Immunology, Division of Hematology Oncology, University of Pittsburgh Cancer Institute, Pittsburgh, Pennsylvania, 15213, USA; 5 Tumor Vaccine Group, Center for Translational Medicine in Women's Health, University of Washington, Seattle, Washington, 98195, USA; 6 Earle A Chiles Research Institute, Robert W Franz Research Center, Providence Portland Medical Center, and Department of Molecular Microbiology and Immunology, Oregon Health and Science University, Portland, Oregon, 97213, USA; 7 Department of Medicine, Division of Hematology, Stanford University, Stanford, California, 94305, USA; 8 Cancer Vaccine Section, NCI, NIH, Bethesda, Maryland, 20892, USA; 9 Discovery Medicine-Oncology, Bristol-Myers Squibb Inc., Princeton, New Jersey, USA; 10 Laboratory of Human Carcinogenesis, Center of Cancer Research, NCI, NIH, Bethesda, Maryland, 20892, USA; 11 Department of Frontier Surgery, Graduate School of Medicine, Chiba University, Chiba, Japan; 12 Baylor Institute for Immunology Research and Baylor Research Institute, Dallas, Texas, 75204, USA; 13 Department of Surgery, Graduate School of Medicine, Osaka University, Osaka, Japan; 14 Section of Surgery, Biomedical Research Unit, Nottingham Digestive Disease Centre, University of Nottingham, NG7 2UH, UK; 15 Centre de la Reserche des Cordeliers, INSERM, Paris Descarte University, 75270 Paris, France; 16 Sapporo Medical University, School of Medicine, Sapporo, Japan; 17 Melanoma Clinic, University of California, San Francisco, California, USA; 18 Department of Molecular Medicine, Sapporo Medical University, School of Medicine, Sapporo, Japan; 19 Division of Cellular Signaling, Institute for Advanced Medical Research, Keio University School of Medicine, Tokyo, Japan; 20 Cellular Technology Ltd, Shaker Heights, Ohio, 44122, USA; 21 Department of Molecular Pathology and Microbiology, Center for Applied Proteomics and Molecular Medicine, George Mason University, Manassas, Virginia, 10900, USA; 22 Illman Cancer Center, University of Pittsburgh, Pittsburgh, Pennsylvania, 15213, USA; 23 Medical Oncology and Immunotherapy, Department. of Oncology, University, Hospital of Siena, Istituto Toscano Tumori, Siena, Italy; 24 Cancer Bioimmunotherapy Unit, Department of Medical Oncology, Centro di Riferimento Oncologico, IRCCS, Aviano, 53100, Italy; 25 Laboratory of Cell Mediated Immunity, SAIC-Frederick, Inc. NCI-Frederick, Frederick, Maryland, 21702, USA; 26 Department of Oncology-Pathology, Karolinska Institute, Stockholm, 171 76, Sweden; 27 The Biomarkers Consortium (BC), Public-Private Partnership Program, Office of the Director, NIH, Bethesda, Maryland, 20892, USA; 28 Department of Cancer Vaccine, Department of Immuno-gene Therapy, Mie University Graduate School of Medicine, Mie, Japan; 29 Department of Medicine, Jonsson Comprehensive Cancer Center, UCLA, Los Angeles, California, 90095, USA; 30 Unit of Immunotherapy of Human Tumors, IRCCS Foundation, Istituto Nazionale Tumori, Milan, 20100, Italy; 31 Department of Pathology, Sapporo Medical University School of Medicine, Sapporo, Japan; 32 Department of Surgery, Division of Surgical Oncology, University of Virginia School of Medicine, Charlottesville, Virginia, 22908, USA; 33 Cancer Therapy Evaluation Program, DCTD, NCI, NIH, Rockville, Maryland, 20892, USA; 34 Cell Therapy Section (CTS), Department of Transfusion Medicine, Clinical Center, NIH, Bethesda, Maryland, 20892, USA; 35 Department of Surgery, Keio University School of Medicine, Tokyo, Japan; 36 Department of Biochemistry, Sapporo Medical University, School of Medicine, Sapporo, Japan; 37 Department of Epidemiology, University of Texas, MD Anderson Cancer Center, Houston, Texas, 77030, USA; 38 Immuneering Corporation, Boston, Massachusetts, 02215, USA; 39 Biometric Research Branch, NCI, NIH, Bethesda, Maryland, 20892, USA; 40 DanDritt Biotech A/S, Copenhagen, 2100, Denmark

## Abstract

Supported by the Office of International Affairs, National Cancer Institute (NCI), the "*US-Japan Workshop on Immunological Biomarkers in Oncology*" was held in March 2009. The workshop was related to a task force launched by the International Society for the Biological Therapy of Cancer (iSBTc) and the United States Food and Drug Administration (FDA) to identify strategies for biomarker discovery and validation in the field of biotherapy. The effort will culminate on October 28^th ^2009 in the *"iSBTc-FDA-NCI Workshop on Prognostic and Predictive Immunologic Biomarkers in Cancer"*, which will be held in Washington DC in association with the Annual Meeting. The purposes of the US-Japan workshop were a) to discuss novel approaches to enhance the discovery of predictive and/or prognostic markers in cancer immunotherapy; b) to define the state of the science in biomarker discovery and validation. The participation of Japanese and US scientists provided the opportunity to identify shared or discordant themes across the distinct immune genetic background and the diverse prevalence of disease between the two Nations.

Converging concepts were identified: enhanced knowledge of interferon-related pathways was found to be central to the understanding of immune-mediated tissue-specific destruction (TSD) of which tumor rejection is a representative facet. Although the expression of interferon-stimulated genes (ISGs) likely mediates the inflammatory process leading to tumor rejection, it is insufficient by itself and the associated mechanisms need to be identified. It is likely that adaptive immune responses play a broader role in tumor rejection than those strictly related to their antigen-specificity; likely, their primary role is to trigger an acute and tissue-specific inflammatory response at the tumor site that leads to rejection upon recruitment of additional innate and adaptive immune mechanisms.

Other candidate systemic and/or tissue-specific biomarkers were recognized that might be added to the list of known entities applicable in immunotherapy trials. The need for a systematic approach to biomarker discovery that takes advantage of powerful high-throughput technologies was recognized; it was clear from the current state of the science that immunotherapy is still in a discovery phase and only a few of the current biomarkers warrant extensive validation. It was, finally, clear that, while current technologies have almost limitless potential, inadequate study design, limited standardization and cross-validation among laboratories and suboptimal comparability of data remain major road blocks. The institution of an interactive consortium for high throughput molecular monitoring of clinical trials with voluntary participation might provide cost-effective solutions.

## Background

The International Society for the Biological Therapy of Cancer (iSBTc) launched in collaboration with the USA Food and Drug Administration (FDA) a task force addressing the need to expeditiously identify and validate biomarkers relevant to the biotherapy of cancer [1]. The task force includes two principal components: a) validation and application of currently used biomarkers; b) identification of new biomarkers and improvement of strategies for their discovery. Currently, biomarkers are either not available or have limited diagnostic, predictive or prognostic value. These limitations hamper, in turn, the effective conduct of biotherapy trials not permitting optimization of patient selection/stratification (lack of predictive biomarkers) or early assessment of product effectiveness (lack of surrogate biomarkers). These goals were summarized in a preamble to the iSBTc-FDA task force [1]; the results are going to be reported on October 28^th ^at the *"iSBTc-FDA-NCI Workshop on Prognostic and Predictive Immunologic Biomarkers in Cancer"*, which will be held in Washington DC in association with the Annual Meeting [2]; a document summarizing guidelines for biomarker discovery and validation will be generated. Several other agencies will participate in the workshop including the National Cancer Institute (NCI), the National Institutes of Health (NIH) Center for Human Immunology (CHI) and the National Institutes of Health Biomarker Consortium (BC).

With the generous support of the Office of International Affairs, NCI, the "*US-Japan Workshop on Immunological Molecular Markers in Oncology*" included, on the US side, significant participation of the iSBTc leadership, representatives from Academia and Government Agencies, the FDA, the NCI Cancer Diagnosis Program (CDP), the Cancer Therapy and Evaluation Program (CTEP), the Cell Therapy Section (CTS) of the Clinical Center, and the CHI, NIH. The participation of Japanese and US scientists provided the opportunity to identify shared or discordant themes across the distinct immunogenetic background and the diverse disease prevalence of the two Nations and compare scientific and clinical approaches in the development of cancer immunotherapy.

Primary goal of the workshop was to define the status of the science in biomarker discovery by identifying emerging concepts in human tumor immune biology that could predict responsiveness to immunotherapy and/or explain its mechanism(s). The workshop identified recurrent themes shared by distinct human tumor models, independent of therapeutic strategy or ethnic background. This manuscript is an interim appraisal of the state of the science and advances broad suggestions for the solutions of salient problems hampering discovery during clinical trials and summarizes emerging concepts in the context of the present literature (Table 1). We anticipate deficiencies in our attempt to fairly and comprehensively portray the subject. However, through Open Access, we hope that this interim document will attract attention. We encourage feed back from readers in preparation of an improved and comprehensive final document [2]. Thus, we invite comments that can be posted directly in the *Journal of Translational Medicine *website and/or interactive discussion through *Knol *[3].

**Table 1:** Emerging biomarkers potentially useful for the immunotherapy of cancer

***Biomarker***	***Therapy***	***Disease***	***References***
***Predictive biomarkers***			
*Telomere length*	Adoptive therapy	Melanoma	[8]
*VEGF*	IL-2 therapy	Melanoma	[9]
*CCR5 polymorphism*	IL-2 therapy	Melanoma	[161]
*Carbonic Anhydrase IX*	IL-2 therapy	Renal Cell Cancer	[267,268]
*IFN-γ polymorphism*	Immuno (IL-2)-chemo	Melanoma	[240]
*STAT-1, CXCL-9, -10, -11, ISGs*	IFN-α therapy	Several Cancers	[182,183]
*IL-1α,-1β, IL-6, TNF-a, CCL3, CCL4*	IFN-α therapy	Melanoma	[262]
*CCL5, CCL11, IFN-γ, ICOS, CD20*	GSK/MAGE3 vaccine	Melanoma	[11,12]
*IL-6 polymorphism*	BCG vaccine	Bladder Cancer	[259]
*MFG-E8*	GM-CSF/GVAX (pre-clin)	Prostate	[273,274]
*T regulatory cells*	hTERT pulsed DCs	Solid Cancer	[275]
*K-ras mutation*	Cetuximab	Colorectal Cancer	[10]
*CCL2, -3, -4, -5 CXCL-9, -10*	Preclinical	Melanoma	[160]
*T cell mulifunctionality*	Preclinical	-	[41]
*SNAIL*	Preclinical	-	[43]
			
***Prognostic Biomarkers (useful for patient stratification/data interpretation)***			
*Oncotype DX, Mamma Print*	-	Breast Cancer	[13,14]
*TGF-β*	-	Breast Cancer	[34]
*Korn Score*	-	Prostate Cancer	[15]
*IFN-γ, IRF-1, STAT-1, ISGs, IL-15, CXCL-9, -10, -11 and CCL5*	-	Prostate Cancer	[254,255]
*IFN-γ, IRF-1, STAT-1*	-	Colorectal Cancer	[134]
*VEGF*	-	Colorectal Cancer, Nasopharyngeal Ca	[141,207]
*ARPC2, FN1, RGS1, WNT2*		Melanoma	[195-197]
			
***Mechanistic/End Point Biomarkers***			
*IFN-γ, IRF-1, STAT-1, ISGs, IL-15, CXCL-9, -10, -11 and CCL5*	IL-2 therapy/TLR-7 therapy	Melanoma/Basal Cell Cancer	[121,126,21]
*IRF-1, STAT-1, ISGs, IL-15, CXCL-9, -10, -11 and CCL5*	Vaccinia virus (Xenografts)	Solid tumors	[137]
*CXCL-9, -10*	Herpes simplex virus (syngeneic model)	Ovarian CA	[166]
*18F-FDG localization*	Anti-CTLA-4 therapy	Melanoma	[102]
*Epitope Spreading*	DC-based therapy	Melanoma	[36]
*Kinetic regression/growth model*	-	-	[24]

## Overview

### Semantics

Howard Streicher (CTEP, Bethesda, MD, USA) presented an overview of biomarkers useful for patient selection, eligibility, stratification and immune monitoring. CTEP sponsors more than 150 protocols each year across many types of new agents, so that this program is familiar with the need to prioritize trials selection using biomarkers. Biomarkers are important for 1) patient selection and stratification for the best therapy; 2) identification of the most suitable targets of therapy; 3) measurement of treatment effect; 4) identification of mechanisms of drug action; 5) measurement of disease status or disease burden and; 6) identification of surrogate early markers of long-term treatment benefit [1].

Examples of biomarkers predictive of immunotherapy efficacy (predictive classifiers) [4-7] are telomere length of adoptively transferred tumor infiltrating lymphocytes which is significantly correlated with likelihood of clinical response [8], serum levels of vascular endothelial growth factor (VEGF), which are negatively associated with response of patients with melanoma to high dose interleukin (IL)-2 administration [9] or K-ras mutations that predict ineffectiveness of cetuximab for the treatment of colorectal cancer [10]. Recently, the European Organization for Research and Treatment of Cancer (EORTC) reported a signature derived from pre-treatment tumor profiling that is predictive of clinical response to GSK/MAGE-A3 immunotherapy of melanoma. The signature includes the expression of CCL5/RANTES, CCL11/Eotaxin, interferon (IFN)-γ, ICOS and CD20 [11,12].

Prognostic biomarkers assess risk of disease progression independent of therapy and can be used for patient stratification according to likelihood of survival thus simplifying subsequent interpretation of clinical results; examples include transcriptional signatures such as Oncotype DX or Mamma Print to stratify breast cancer patients [13] though their usefulness needs further validation [14]. Korn et al [15] proposed the incorporation of multivariate predictors such as performance status, presence of visceral or brain disease and sex to interpret correlations between response and survival data in early-phase, non-randomized clinical trials. Similarly, body mass and other parameters could predict individual survival probabilities and help stratify patients with prostate cancer in randomized phase III trials [16]. Recently, Grubb et al. [17] described a signaling proteomic signature based on a comprehensive analysis of protein phosphorylation that could be used for the stratification of patients with prostate cancer. Guidelines for the identification of potential classifiers during explorative, high throughput, discovery-driven analyses were proposed by Dobbin at al. [18]; they include the assessment of 3 parameters: standardized fold change, class prevalence, and number of genes in the platform used for investigation. Assessment is based on an algorithm that guides the determination of the adequacy of sample size in a training set. A web site is available to assist in the calculations [19].

Analyses performed during or right after treatment can provide mechanistic explanations of drugs function such as the intra-tumor effects of systemic interleukin (IL)-2 therapy [20] or local application of Toll-like receptor agonists [21] (mechanistic biomarkers). End point biomarkers assure that the expected biological goals of treatment were reached. Best examples are the immune monitoring assays performed during active specific immunization [22,23]. Surrogate biomarkers inform about the effectiveness of treatment in early phase assessment and help go/no go decisions about further drug development [1]. This is important because tumor response rates documented during phase II trials have not been, with few notable exceptions, reliable indicators of meaningful survival benefit. The series of phase II trials of cooperative group studies in North America over the past 35 years have shown little evidence of impact for single agents, but have identified benchmarks of outcome that now may be addressed, including progression at 6 months (18%), and survival at 12 months (25%) that have been unaltered over the interval of the study. These benchmarks may now allow us to accelerate progress by developing adequately powered phase II studies that would serve as the threshold for decision making for new phase III trials [15]. Recently, a new survival prediction algorithm was proposed; tumor measurement data gathered during therapy are extrapolated into a two phase equation estimating the concomitant rate of tumor regression and growth. This kinetic regression/growth model estimates accurately the ability of therapies to prolong survival and, consequently, assist as a surrogate biomarker for drug development [24].

### Steps in biomarker discovery

Since the term "biomarker" is used for a wide variety of purposes, confusion often results when biomarker development, validation and qualification are discussed [7,25,26]. During phase I and II clinical trials that are meant to establish dose, schedule and drug activity, biomarkers should primarily show biological effect of the drug (i.e. demonstrate whether a drug reached its target) and do not need to be validated as a surrogate equivalent of long term benefit. As the drug assessment process proceeds the expectations of a given biomarker grow in parallel. Moving from correlative science to clinically applicable biomarkers, validation of the marker and the assay in cohorts need to be performed. At this stage, it is important to separate data used to develop classifiers from data used for testing treatment effects. The process of classifier development can be exploratory, but the process of evaluating treatments should not be. Ultimately, clinical qualification of the marker for clinical use should be based on testing specific hypotheses in prospectively selected patient populations.

This was emphasized by Nora Disis (University of Washington, Seattle, WA, USA) who discussed steps in biomarker validation [27]. Referring to work from Pepe et al [28-31], five phases of biomarker development were described: 1) pre-clinical exploratory phase that identifies promising directions; 2) clinical validation in which an assay can detect and characterize a disease; 3) retrospective longitudinal validation (i.e. a biomarker can detect disease at an early stage before it becomes clinically detectable or has other predictive value); 4) prospective validation of the biomarker accuracy and 5) testing its usefulness in clinical applications to predict clinically relevant parameters. An example of exploratory studies is the identification of a distinct phenotype of functional T cell responses and cytokine profiles that distinguish immune responses to tumor antigens in breast cancer patients [32]. Tumor antigen-specific immune responses in cancer patients were observed to differ from responses to common viruses. In particular, a reduced frequency of IFN-γ-producing CD4 T cells was observed. In this discovery phase, it may be useful to test pre-clinical models to verify the strength of an hypothesis [33]. Following the steps of validation, a retrospective analysis suggested that survival is associated with development of memory immune responses [34] or that changes in serum transforming growth factor (TGF)-β values are prognostic in breast cancer; an inverse correlation between TGF-β levels and development of immune responses and epitope spreading during immunotherapy was found to be of clinical significance. Similar importance of epitope spreading was previously reported by others in the context of dendritic cell (DC)-based immunization against melanoma [35-38] or antigen-specific, epitope-based vaccination [39]. Important exploratory findings were reported by Hiroyoshi Nishikawa (Mie University, Mie, Japan) [40], who observed a good correlation between antibody and T cell responses following NY-ESO-1 protein vaccine suggesting that cellular immune responses could be extrapolated following the simpler to measure humoral responses. A detection system was developed to identify antibodies against NY-ESO-1 that was validated by inter-institutional cross validation. The assay was tested in patients with esophageal cancer who expressed NY-ESO-1.

### Pre-clinical screening for biomarker identification

Studies in transgenic mice shed insights about the kinetics of activation of vaccine-induced T cells useful for the design of future monitoring studies. DUC18 transgenic mice bearing CMS5 tumors were studied. Adoptive T cell transfer of mERK2-recognizing T cells obtained from mice 2, 4 or 7 days after immunization demonstrated that only those obtained 2 days after immunization could control tumor growth in recipient animals. Cytokine expression analysis suggested that outcome was correlated with the breath of the cytokine repertoire produced by the adoptively transferred T cells (multi-functionality); the multi-functionality was time-dependent and was maximal in T cells harvested 2 days after immunization. Tumor challenge did not restore multi-functionality while ablation of T regulatory cells did. Also peptide vaccination rescued multifunctional T cells *in vivo*. This pre-clinical model suggests that cytokine secretion panels should be included for immune monitoring of patients with cancer [41]. Bernard Fox (Earle A Chiles Research Institute, Portland, OR, USA) presented a model in which the effect of anti-cancer vaccination was tested in conditions of homeostasis-driven T cell proliferation in lymphocyte depleted hosts [42]. Lymphopenia strongly enhanced the expansion of CD44^hi^CD62L^lo ^T cells in tumor vaccine-draining lymph nodes which corresponded to higher anti-cancer protection compared with normal mice. This study suggested that vaccination could be performed during immune reconstitution in immunotherapy trials utilizing immune depletion and that a target T cell phenotype could be used as a potential mechanistic/end point biomarker. When the experiments were repeated in mice with established tumor, depletion of T regulatory cells was required for therapeutic efficacy. The design of their current clinical trial translating finding from preclinical studies was discussed. Yutaka Kawakami (Keio University, Tokyo, Japan) presented an animal model in which SNAIL expression (a gene involved in tumor progression) induced resistance of tumors to immunotherapy (see later) and may represent a new predictive biomarker of tumor responsiveness to immune therapy if validated in humans [43].

### Validation and standardization of current biomarker assays – a link to the iSBTc/FDA task force

Lisa Butterfield (University of Pittsburgh, Pittsburgh, PA, USA) and Nora Disis summarized validation efforts on immunologic assay performance and standardization [22,23,44-49]. This effort is critical to the selection of true biomarkers over the "noise" of assay variation in order to have reliable, standardized measures of immune response. This is a primary focus of one of the two "iSBTc-FDA Taskforce on Immunotherapy Biomarkers" working groups. Published guidelines for blood shipment, processing, timing and cryopreservation were presented together with examples of standardization of the most commonly used immune response assays; the IFN-γ ELISPOT, intra-cellular cytokine staining and major histocompatiblity multimer staining [45]. Understanding the cryobiology principles that explain cellular function after preservation is becoming extremely important as multi-institutional studies require shipment of specimens across vast distances often following non-standardized procedures. Recent studies illustrate the potential for improving the cryopreservation of stem cells. Standardization of cell processing has led to the study of liquid storage prior to cryopreservation, validation of mechanical (uncontrolled rate freezing) freezing, and cryopreservation bag failure [50,51].

Extensive discussion about assay validation is beyond the purpose of this report as it was discussed in the previous related manuscript [1]. However, it is important to emphasize the proven need for assay standardization with standard operating procedures utilized by trained technicians (who undergo competency testing), the need for standard and tracked reagents and controls, and more broadly accepted, shared protocols which would allow for better cross-comparisons between laboratories. The guidelines of CLIA (Clinical Laboratory Improvements Amendments), which include definitions of test accuracy, precision, and reproducibility (intra-assay and inter-assay) and definitions of reportable ranges (limits of detection) and normal ranges (pools of healthy donors, accumulated patient samples) are available at the CLIA website [52]. Butterfield included examples of assay standardization performed at the University of Pittsburgh Immunologic Monitoring and Cellular Products Laboratory. A good example is the development of potency assays for the maturation of DCs; recently production of IL-12p70 was shown to represent a useful marker that could distinguish between DC obtained from normal individuals compared to those obtained from individuals with cancer or chronic infections [53], a similar consistency analysis was reported by others [54]. Use of central laboratories may help overcome the extensive cost and effort of this level of standardization [46,55].

### The Biomarkers Consortium (BC): *A Novel Public-Private Partnership Leading the Cutting-edge of Biomarkers Research*

Although not active participant in the workshop, the NIH BC deserves mention because it purposes converge toward the issue discussed herein and future efforts in biomarker discovery should taken into account the potential usefulness of this NIH initiative. The promise of biomarkers as indicators to advance and revolutionize many aspects of medicine has become a reality for researchers in all sectors of biomedical research. Biomarkers include molecular, biological, or physical characteristics that indicate a specific, underlying physiologic state to identify risk for disease, to make a diagnosis, and to guide treatment [56]. Given the breadth of utility of biomarkers, the importance of cross-sector and cross-therapeutic research efforts is inevitable and the BC has taken a first step to implement this reality. The BC is a unique partnership among FDA, NIH and Industry, serving the individual missions of each organization while focusing on biomarkers, an area of alignment of the interests of all the consortium's participants. The mission of the BC is to brings together the expertise and resources of various partners to rapidly identify, develop, and qualify potential high-impact biomarkers. The Consortium's founding partners are the NIH, the FDA, and Pharmaceutical Research and Manufacturers of America (PhRMA). Additional partners represent Center for Medicare and Medicaid Services, biopharmaceutical companies and trade organizations, patient and professional groups, and the public, and partners in all categories share a common goal- using biomarkers to hasten the development and implementation of effective interventions for health and fighting disease. The BC was formally launched in late 2006 to identify and qualify new, quantitative biological markers ("biomarkers"), for use by biomedical researchers, regulators and health care providers. Effective identification and deployment of biomarkers is essential to achieving a new era of predictive, preventive and personalized medicine. Biomarkers promise to accelerate basic and translational research, speed the development of safe and effective medicines and treatments for a wide range of diseases, and help guide clinical practice. The BC endeavors to discover, develop, and qualify biological markers or "biomarkers" to support new drug development, preventive medicine, and medical diagnostics.

Operations of the BC are managed by the Foundation for the NIH (FNIH), a free-standing charitable foundation with a congressionally-mandated mission to support the research mission of the NIH. As managing partner, the FNIH is responsible for coordinating both the funding and administrative aspects of the BC and staffs the executive committee, steering committee and project team members with respect to BC operations.

The Biomarkers Consortium is creating fundamental change in how healthcare research and medical product developments are conducted by bringing together leaders from the biotechnology and pharmaceutical industries, government, academia, and non-profit organizations to work together to accelerate the identification, development, and regulatory acceptance of biomarkers in four key areas: cancer, inflammation and immunity, metabolic disorders, and neuroscience. Results from projects implemented by the consortium will be made available to researchers worldwide.

### The special case of array technology – A balance in reproducibility, sensitivity and specificity of genes differentially expressed according to microarray studies

A discussion about biomarkers relevant to the clinics warrants special attention to high-throughput technologies and, among them, the use of global transcriptional analysis platforms [57,58]. Indeed, in the last decade, microarray technology has arguably offered the most promising tool for discovery-driven, patient-based analyses and, consequently, for biomarker discovery [59]. Several publications claimed that microarrays are unreliable because list of differentially expressed genes are often not reproducible across similar experiments performed at different times, with different platforms, and by different investigators. The FDA has taken leadership in testing such hypothesis through the MicroArray Quality Control (MAQC) project whose salient results have been recently summarized [57,60]. Comparisons using same microarray platforms and between microarray results were performed and validated by quantitative real-time PCR. The data demonstrated that discordance between results simply results from ranking and selecting genes solely based on statistical significance; when fold change is used as the ranking criterion with a non-stringent significant cutoff filtering value, the list of differentially expressed genes is much more reproducible suggesting that the lack of concordance is most frequently due to an expected mathematical process [57]. Moreover, comparison of identical sample expression profile performed on different commercial or custom-made platforms at different test sites yielded intra-platform consistency across test sites and high level of inter-platform qualitative and quantitative concordance [58,61]. Quantitative analyses of gene expression comparing array data with other quantitative gene expression technologies such as quantitative real-time PCR demonstrated high correlation between gene expression values and microarray platform results [62]; discrepancies were primarily due to differences in probe sequence and thus target location or, less frequently, to the limited sensitivity of array platforms that did not detected weakly expressed transcripts detectable by more sensitive technologies. The conclusion, however, was that microarray platforms could be used for (semi-)quantitative characterization of gene expression. When one-color to two color platforms were compared for reproducibility, specificity, sensitivity and accuracy of results, good agreement was observed. The study concluded that data quality was essentially equivalent between the one- and two-color approaches suggesting that this variable needs not to be a primary factor in decisions regarding experimental microarray design [63].

Raj Puri (FDA, Bethesda, MD, USA), suggested that, the consistency and robustness of high throughput technology, particularly, in the area of transcriptional profiling can be used to evaluate product quality particularly when tissue, cells or gene therapy products are proposed for clinical utilization and potential licensing; these materials may display a consistent phenotype based on standard markers but display different genetic characteristics when examined at the global level. Several examples are emerging that may affect the interpretation of data on cellular products adoptively transferred to patients. David Stroncek (CTS, NIH, Bethesda, Maryland, USA) [64] showed that different maturation schemes of DCs or stem cells bear quite different results in their transcriptional phenotype even when similar agents are used [65-68]. Similar work has been reported by the FDA on stem cell characterization [69-71]; same principles were followed to address assay reproducibility in freeze and thaw cycles [72] or changes in culture conditions [73]. By using this validation approaches it will be hopefully possible to enhance the quality of potency assessment for cellular products [64]; this will provide consistency across clinical protocols performed in different institutions and may facilitate identification of novel clinically-relevant biomarkers. With this purpose, the FDA as developed a web site offering guidance for pharmacogenomic data submission [74-76].

## Novel monitoring approaches

### Monitoring of tumor specific immune responses to undefined antigens

Some vaccine-therapies target whole proteins or cell extracts which have the advantage of exposing the immune system to a broader antigenic repertoire. However, it is difficult to verify whether antigen-specific responses were elicited by the vaccine since the relevant antigen is often not known. For instance, the utilization of GVAX against prostate follows surrogate end points such as prostate-specific antigen levels or doubling time [77]. However, it is difficult to characterize the immune response because strong allo-reactions are generated by the foreign cancer cells and no clear antigen relevant to the autologous tumor is known. Thus, monitoring strategies need to be designed for these situations. Fox suggested the screening of pre- and post-vaccination sera looking for developing antibodies. This could be done with commercially available protein arrays that allow screening of thousand of proteins. Indeed, increased prostate-specific antigen doubling time correlates with immune responses toward a limited number of tumor-associated antigens. At the same time, T cell responses can be monitored following antigen presentation by autologous antigen presenting cells fed with proteins identified by the analysis of sera on protein arrays. Since it is unknown whether the immune responses are targeting antigens expressed by vaccine, but not tumor, circulating tumor cells might be used to examine whether specific antigens were expressed by tumor.

Anti cytotoxic T lymphocyte antigen (CTLA)-4 antibodies have been used in hundreds of patients confirming a low but reproducible response rate of about 10%. Most responses, however, are long term and 20 to 30% are associated with severe autoimmune toxicities. There is a critical need to understand the mechanism(s) leading to response and/or toxicity. Antoni Ribas (UCLA, Los Angeles, CA, USA) described the characterization of immune responses during anti-CTLA-4 therapy. Following guidelines to define assay accuracy as suggested by Fraser [78,79], careful analyses were performed taking into account technical (different protocols), analytical (same procedure, variations in replicates) and physiological (same person, different results over time) sources of variance. A true response was defined as a value above the Mean+3SD normal controls [80,81]. With these stringent criteria, neither expansion nor decrease in circulating T regulatory cells supposed to be primary targets of the treatment was observed. However, post-treatment gene expression profiling demonstrated activation of T cells. Phospho-flow assays using cellular bar-coding, which allows multiplex analysis of different cell subsets suggested that tremelimumab induces activation of pLck, phosphorylated signal transducer and activator of transcription (STAT)-1 in CD4 cells while phosphorylation of STAT-5 decreases. Moreover, a decrease in phospho Erk was observed in both CD4+ and CD14+ cells. Surprisingly, the therapy affected monocytes not previously known to be targets of anti-CTLA-4 therapy. However, subsequent analyses demonstrated that monocytes express CTLA-4 emphasizing the importance to study the immune responses at a multi-factorial and unbiased level [82-84]. In addition, an increase in IL-17-expressing CD4 T cells was observed after treatment that correlated with autoimmune toxicity and inflammation although no direct correlation with clinical response was noted [85].

### Novel cytotoxicity assays

Cell specific assays based on ELISPOT technology or FACS analysis are emerging that directly or indirectly characterize cell capability to carry effector functions. This is important because dissociations have been described between cytokine and cytotoxic molecule expression [86-88]. ELISPOT assays that detect the effector response of cytotoxic T cells to cognate stimulation have been recently described [89-91]. More recently, a flow cytometric cytotoxicity assay was developed for monitoring cancer vaccine trials [92]. The assay simultaneously measures effector cell de-granulation and target cell death. Interestingly, as previously shown using transcriptional analyses and target cell death estimation [86], this assay demonstrated that vaccine-induced T cells in patients undergoing vaccination with the gp100 melanoma antigen do not display cytotoxic activity *ex vivo *but the cytotoxic activity could be restored by *in vitro *antigen recall. These observations are supported also by others findings that IFN-γ and granzyme-B production by recently activated CD8+ memory T cells fades few days after stimulation as the immune response contracts into the memory phase [86,93-95]. Thus, future monitoring trials should include a broader range of assays testing the expression/secretion of different cytokines and cytotoxic molecules.

### Imaging technologies to study trafficking

There are several examples of differences between therapy-induced changes in the tumor microenvironment compared with the peripheral circulation [20,96-98]. Ribas, proposed the study of the kinetics of anti-tumor immune responses *in vivo *using PET-based molecular imaging [99] expanding the analysis of immune conjugate kinetics for pharmacokinetics studies and visualization of lymphoid organs [100,101]. Tools to evaluate the function of lymphoid tissue or other components of the tumor microenvironment are critical to assess the dynamic of response to anti-CTLA4 therapy and, likely, other forms of immunotherapy. Tumors do not decrease in size and may even increase due to inflammation and necrosis in the early phases of anti-ACTL-4 treatment and, therefore, tumor size is not a reliable predictor of response. However, 18F-FDG was a useful early marker of response demonstrating increased glycolitic activity by activated immune cells [102].

## Proteomic approaches

### High throughput reverse phase protein microarrays (RPMA) for signal pathway profiling

Global profiling of protein activation is an important tool for the understanding of the signaling response to immune stimulation. Julia Wulfkuhle (George Mason University, VA, USA) described novel proteomics approaches that could be particularly useful for immune monitoring.

A clear example is the complexity of the response to type I IFNs. It is becoming increasingly appreciated that signaling down-stream of type I IFNs is more complicated than predicted by the reductionist Jak/STAT model [103,104]. In highly controlled experimental settings we could not demonstrate a direct quantitative relationship between STAT-1 phosphorylation and activation of interferon-stimulated genes (ISGs) (Pos et al. manuscript in preparation); a deeper characterization of interactions among STAT dimers [105] and among alternative pathways is necessary to fully understand the mechanisms of IFN-induced responses and their relationship with TSD [103]. RPMA provide the opportunity to study the phosphorylation states of hundreds of signaling molecules at the same time and potentially provide better characterization of the mechanisms controlling downstream transcription following cytokine stimulation [17,106-108]. Although most studies performed with these arrays were limited to the understanding of transformed cell biology, it is possible to apply these technologies to cellular subsets obtained from the peripheral circulation or from tumor tissues during immunotherapy trials. While the RPMA technology allows for the analysis of hundred of proteins at the time, it is not cell-specific and special precautions in the preparation of samples are necessary such as laser capture microdissection or cell sorting for single cell populations. Gary Nolan's group at Stanford, has developed a conceptually similar approach for the study of signaling pathways at the cellular level that utilized multi-color FACS analysis [83,109,110]. However, multi-color FACS analysis is limited to the analysis of only a dozen endpoints at once while RPMA analysis provides measurements of 150–200 signaling proteins with the same starting cell number. Either of these approaches is likely to provide comprehensive functional information about the status of activation and responsiveness of immune cells during immunotherapy.

### Tissue handling processing can affect the status of phosphoproteins – novel molecular fixatives

Following procurement the tissue remains alive and is subject to hypoxic and metabolic stress while being transported or reviewed by the pathologist prior to freezing or formalin fixation. Time taken to obtain and preserve material, concentration of endogenous enzymes, tissue thickness and penetration time, storage temperature, staining and preparation; all of these factors can directly affect the phosphorylation status of a protein [111] and the expression of the protein as well as messenger RNA levels [112]. During the delay time prior to molecular stabilization the kinase pathways are active and reactive. Consequently, in order to stabilize phosphoproteins during the pre-analytical period it is necessary to inhibit the activity of kinases as well as phosphatases. Use of permeability enhancers can potentially change the speed of tissue phosphoproteins activation and phosphatase and kinase inhibitors can stop this process ; these novel fixatives are becoming commercially available.

### Biomarker harvesting using nano-particles

"Smart" core shell affinity bait nano-porous particles amplify the concentration of a given analyte [113]. The analyte molecule binds to high affinity bait inside the particle. The analyte is concentrated because all of the target analyte is removed from the bulk solution and concentrated in the small volume of nanoparticles. Concentration factors can excide 100 fold. Different chemical "baits" are used to capture different kind of proteins based on charge or other biochemical characteristics. The size of the nanoparticles shell pores determines the protein size cutoff that can enter the particle. Biomarkers, chemokines or cytokines can be separated from larger proteins present at much higher concentrations. In addition, the binding to the bait stabilizes the captured analyte protein against degradative enzymes. This approach may be particularly useful for the study of serum cytokines which are, even at bioactive levels, at concentrations below the threshold of detection of most non antibody-based methods [114,115].

### Computational Approaches

Computational models of the immune system can provide additional tools for understanding and predicting response to immunotherapy. Doug Lauffenburger developed a set of mechanism-based models to predict *in vitro *behavior of immune system cells through a quantitative analysis of receptor-ligand binding and trafficking dynamics [116]. Extending this approach to clinical applications, Immuneering Corporation is developing modeling technology to analyze measurements taken from patient samples, and preparing proof of concept trials to assess the responsiveness of melanoma and renal cell carcinoma patients to IL-2 therapy. Advanced techniques for the validation of computational models have also been developed [117]. Among them, the modular analysis of disease-specific transcriptional patterns developed by Chaussabel et al [118,119] holds promise to represent an important tool to comprehensively follow the modulation of immune responses during therapy (see later).

## Emerging concepts in biomarker discovery; the state of the science

### Signatures from the tumor microenvironment

Most presentations by US participants discussed the immune biology of cutaneous melanoma as a prototype of cancer immunotherapy; most Japanese presentations (a Country with limited prevalence of melanoma) discussed other cancers. Thus, while cutaneous melanoma provided a paramount model to discuss cancer immune biology, other cancers offered an overview at potential expansion of emerging concepts to other diseases (*i.e*. common solid cancers) and other ethnic groups (the Asian population) [120]. Though disease- or population-specific patterns were observed, commonalities were identified that support the hypothesis of a constant mechanism that leads to TSD [121].

### From the delayed allergy reaction to the immunologic constant of rejection

In 1969, Jonas Salk suggested that the delayed hypersensitivity reaction of the tuberculin type, contact dermatitis, graft rejection, tumor regression and auto-allergic phenomena such as experimental allergic encephalomyelitis were facets of a single entity that he called "the delayed allergy reaction [122]. Expanding on this argument, we proposed that tumor rejection represents an aspect of a broader phenomenon responsible for TSD that occurs also in autoimmunity, clearance of pathogen-infected cells or allograft rejection [121,123-125]. Transcriptional studies done in humans at the time when tissues transition from a chronic lingering inflammatory process to an acute one leading to TSD point to common mechanisms that are activated during immunotherapy against cancer or chronic viral infections or dampened when inducing tolerance of self in autoimmunity or of allografts in transplantation. This theory emphasizes the need to deliver potent pro-inflammatory stimuli in the target tissue. Antigen-specific effector-target interactions are not sufficient to induce TSD but rather act as triggers to induce a broader activation of innate and adaptive immune responses. Given a conducive microenvironment, these responses can expand to an acute inflammatory process inclusive of several effector mechanisms. Thus, immunotherapy should amplify the inflammatory processes induced by tumor-specific T cells within the tumor microenvironment.

### Interferon-stimulated genes (ISGs) – Some ISGs are more significant than others

Comparisons of transcriptional studies performed by various groups in human tissues undergoing acute (but not hyper-acute) rejection suggests that TSD encompasses at least two separate components: the activation of ISGs and the broader attraction and *in situ *activation of innate and adaptive immune effector functions (IEF) mediated by a restricted number of chemokines and cytokines. While the ISGs are consistently present during rejection, IEFs may vary according to the model system studied. Examples include the acute inflammatory process inducing regression of melanoma metastases during IL-2 therapy [20,126] or basal cell cancer by Toll-like receptor-7 agonists [21]. The same signatures are observed in acute but not in chronic HCV infection leading to clearance of pathogen [127-129] and in acute uncontrollable kidney allograft rejection [130]. Furthermore, activation of ISGs is a classic signature associated with systemic lupus erythematosus and tightly correlates with the severity of the disease [118,131,132]. Moreover, coordinate expression of specific ISGs such as IRF-1 linked with the induction of adaptive Th1 immune responses with genes mediating cytotoxicity and the CXCL-9 through -11 chemokines has been associated with better prognosis in colorectal cancer [133-135]. Interestingly, similar results are observable in experimental mouse models. According to the linear model of T cell activation, ISGs and IEFs activation is short lasting and is rapidly followed by a contraction phase [93]; the signatures associated with the acute phase can be observed within the tumor microenvironment during adaptive and/or innate immunity-mediated tumor regression [136,137].

It should be emphasized that the expression of ISGs is necessary but not sufficient for the induction of TSD as it is observed also in chronic inflammatory processes that do not lead to TSD [121]. However, the definition of ISGs in itself is vague and refers to a large repertoire of genes that may be activated by type I IFNs in various conditions depending upon the type of cell stimulated and the conditions in which the stimulus is provided [138]. Although canonical ISGs (those stimulated by type I IFN) are regularly observed during TSD, it appears that those most specifically associated with TSD but not chronic inflammatory processes are ISGs downstream of IFN-γ stimulation such as interferon-regulatory factor (IRF)-1 [139-141] and STAT-1 [105]. Importantly, IRF-1 specifically promotes IL-15 expression [139], which is central to the induction of TSD [137]. IRF-3 is also commonly activated during TSD; IRF-3 is responsible for the over-expression of CXCL-9 through -11 and CCL5 chemokines [139] which also play a central role in TSD. This signature of acute inflammation are in contrast with the indolent inflammatory process that fosters cancer growth and hampers immune responses [123,142-146]; in particular, the extensive expression of immune-inhibitory mechanisms during tumor progression [147] dramatically contrast with the picture observed during TSD and emphasizes the need to study the tumor microenvironment at relevant moments when the switch from chronic to acute inflammation occurs [148-150].

### Chemokines, cytokines and effector molecules

The comparative approach described so far [124] suggests that TSD is determined by the expression of a limited number of genes generally associated with Th1 immune responses. Among them IL-15 and its own receptors play a central role in clinical and experimental models of tumor rejection [21,137,151]. Together with IL-15 the chemokines CCL5/RANTES and CXCL-9/Mig -10/IP-10 and -11/I-TAC are consistently present during TSD and probably serve as central attractors of CXCR3 and CCR5-expressing effector T and NK cells [152]. In particular, CD8 T cell infiltration to inflamed areas such as the cerebrospinal fluid in multiple sclerosis [153], atherosclerotic plaques [154] or allografts [155,156] is predominantly mediated by CXCR3 ligand chemokines, which also play a central role in tumor rejection. This observation collimates with a recent report suggesting that CXCR3 expression in CTL is associated with survival benefit in the context of melanoma [157]. This finding could be explained by the heavy lymphocyte infiltration present in melanoma metastases expressing of CXCR3 ligand chemokines such as CXCL9/Mig [158] and CXCL10/Ip-10 [159]. A finding recently confirmed by independent investigators [160]. Interestingly, CCL5/Rantes and IFN-γ were also reported to predict immune responsiveness during GSK/MAGE-A3 immunotherapy [12]. Moreover, the role played by CCL5/RANTES is suggested by the weight that CCR5 polymorphism plays in the prognosis of melanoma [161]. More recently, Kalinski et al [162] proposed the utilization of DCs conditioned to drive the development of immune responses toward Th-1 immunity by conditioning DC with a mixture of polycytidylic acid (poly-I:C), IFN-α and IFN-γ. These DCs express CXCR3 and CCR-5 ligands that promote the chemotaxis and *in situ *expansion of effector cytotoxic T cell phenotype. Additionally, these DCs repress the expansion of T regulatory cells since they do not express the CXCR4 ligand chemokine CCL22/MDC [163,164]. Most importantly, these DC can regulate T cell homing properties. This is explained by the three wave model of myeloid and plasmacytoid DC production of chemokines [165]; upon viral stimulation, DC secrete in the first 2 to 4 hours chemokines potentially attracting a broad range of innate and adaptive effectors cells such as neutrophils, cytotoxic T cells, and natural killer cells (CXCL1/GROα, CXCL2/GORβ, CXCL3/GROγ and CXCL16); in a second phase lasting between 8 and 12 hours, they secrete chemokines that attract activated effector memory T cells (and to a lesser degree NK cells) (CXCL8/IL-8, CCL3/MIP-1α, CCL4/MIP-1β, CCL5/RANTES, CXCL9/Mig, CXCL10/IP-10 and CXCL11/I-TAC); finally, the third resolving wave occurs 24 to 48 hours following stimulation producing chemokines that attract regulatory T cells (CCL22/MDC) or naïve T and B lymphocytes in lymphoid organs (CCL19/MIP-3β and CXCL13/BCA-1). Possibly, the intensely pro-inflammatory IFN and poly-I:C-based conditioning prolongs the acute phase of DC activation and the same may occur *in vivo *during the acute inflammatory process leading to TSD.

Pre-clinical models also clearly underline the central role that CXCR3 ligand chemokines play in recruiting activated effector T cells and NK cells at the tumor site. In particular, oncolytic viral therapy was recently shown to induce powerful anti-cancer immune responses that are centrally mediated by CXCL-9/Mig, -10/IP-10, -11/I-TAC and CCL5/RANTES. Similar results were obtained delivering oncolytic herpes simplex virus in a syngeneic model of ovarian carcinoma [166] or by the systemic administration of vaccinia virus colonizing selectively human tumor xenografts [137].

### Location, orientation and organization of the immune infiltrates

Jérôme Galon, Franck Pagès, Marie-Caroline Dieu-Nosjean and Wolf-Hervé Fridman have analyzed the immune infiltrates in large cohorts of colorectal and non small cell lung cancers. High densities of T cells with a TH1 orientation and high numbers of CD8 T cells expressing perforin and granulysin, enumerated at the time of surgery, appear to be the strongest prognostic factor (above TNM staging) for disease free and overall survival, at all stages of the disease [133,134]. Genes associated with adaptive immunity (i.e. CS3, ZAP70) TH1 orientation (i.e. T-bet, IFNγ, IRF-1) and cytotoxicity (i.e. CD8, granulysin) correlated with low levels of tumor recurrence whereas that of genes associated with inflammation or immune suppression did not [134]. The immune responses needed to be coordinated both in terms of location (center of the tumor and invasive margin (2)) and of orientation with memory and TH1 but not TH2, lack of immune suppression, and in terms of inflammation or angiogenesis [167]. Moreover, in the few patients with high T cells infiltration who presented with metastasis at the time of diagnosis, there was a loss of effector/memory T cells in the tumor [141]. Adjacent to the tumors, some patients presented with tertiary lymphoid structures containing germinal center – like structures composed of mature dendritic cells, CD4 and CD8 lymphocytes and activated B cells, a likely place for a local immune reaction to be generated [168]. This finding supports a potential helper role that B cells may play in the recruitment and activation of effector T cells [169]. The resemblance of tertiary lymph nodes were particularly evident in early stage cancers [133,168] and the enumeration of memory TH1 (IFNγ-producing) and CD8 (granulysin producing) T cells in the center and invasive margin of human tumors should become part of the prognostic setting of human tumors [167,170]. This recommendation is also based on concordant observations extended to several other tumors [171-176].

### Signatures from circulating immune cells and soluble factors

Bernard Fox emphasized the need for a comprehensive approach to the characterization of immune responses that trespasses the simple enumeration of tumor antigen-specific T cells. Characterization by 8 color flow cytometry of vaccine-induced T cells in patients with melanoma vaccinated with the gp100 melanoma antigen demonstrated a wide range of functionality that spanned from different avidity for target antigen, to different levels of tumor-induced CD107 mobilization [177]. Importantly, it was noted that vaccine-induced T cells do not acquire in the memory phase enhanced functional avidity usually associated with competent memory T-cell maturation; these data suggest that other vaccine strategies are required to induce functionally robust long-term memory T cell function [178]. Concordant results have been previously reported by Monsurró et al. [86] by profiling the transcriptional patterns of vaccine-induced memory T cells; a quiescent phenotype was observed that required *in vitro *antigen recall plus IL-2 stimulation to recover full effector function. Similar observations have been also recently reported by others [94,95]. Thus, vaccination is not sufficient to produce effector cells qualitatively and quantitatively capable to induce cell-mediated TSD unless a secondary reactivation is provided at the receiving end by combination therapy [179].

Damien Chaussabel (Baylor Institute for Immunology, Dallas, Texas, USA) summarized his work profiling circulating peripheral blood mononuclear cell (PBMC) adopting a modular analysis framework to reduce the multidimensionality of array data. This strategy enhances the visualization through the reduction of coordinately expressed transcripts into functional units [118,119]. With this approach, PBMCs display a disease-specific pattern; individuals with a given disease bear transcriptional fingerprints that are qualitatively and quantitatively related to the severity of the disease. The modular process has been successfully used to identify patients at high risk for liver transplant rejection. It is interesting that a similar approach was recently described by others to identify patients with HCV infection likely to respond to IFN-α therapy; analysis of PBMC signatures *ex vivo *and their responsiveness to IFN-α stimulation was a predictor or clinical outcome [180]. More recently the Baylor group, in collaboration with John Kirkwood has expanded this approach to the monitoring of patients with melanoma treated by active specific immunization; preliminary observations identified baseline differences among patients and enhancement of IFN-modular activity following treatment.

Immunologic differences between patients with cancer and non-tumor bearing individuals were conclusively confirmed by the work of Peter Lee (Stanford University, Stanford, California, USA) [181,182]; PBMCs from patients with melanoma and other solid cancers [183] display strongly reduced responsiveness to IFN-α stimulation that can be measured by intra-cellular staining for phosphorylated STAT-1 protein. Gene expression profiling of lymphocytes from patients with Stage IV melanoma identified 25 genes differentially expressed in T and B cells of cancer patients compared with carefully selected normal controls; of the 25 genes, 20 were ISGs among which CXCL9–11, STAT-1, OAS and MX-1 were included; all of them are critical component of the immunologic constant or rejection ([121,137] and were down-regulated in cancer patients. The top 10 genes could separate melanoma patients from healthy individuals in self-organizing clustering. Phosphorilation of STAT-1 is a primary component of IFN-signaling and, therefore, a phospho-assay was developed. Originally T cells were found to be predominantly affected but with more cases studied also B cells were recognized as affected [183]. PBMCs from patients with breast cancer demonstrated the same difference in STAT-1, IFI44, IFIT1, IFIT2, and MX1 expression and were similarly unresponsive to IFN-α stimulation. The same results were observed in patient with gastrointestinal cancers where the same effects could be observed in T, B and NK cells. IFN-γ induced phosphorilation is only affected in B-cells, while very little dynamic response is seen in T cells and NK cells. This may be related to a dynamic alteration of IFN-γ receptor in various stages of T cell activation [184]. These alterations appear already at STAGE II of disease and continue as the disease progresses. It is not known whether other signaling defects are present in these cells. This is possible considering the reported alternations of T cell receptor signaling described in the past by others [185-187] and in general altered T cell function in circulating and/or tumor infiltrating lymphocytes [86,147,179,187]. Indeed, also in Lee's study a decrease in expression of CD25, HLA-DR, CD54 and CD95 was observed. Most recently, STAT-1 phosphorylation analysis was applied to patients undergoing immunotherapy with high-dose IFN-α and preliminary results suggest that responding patients display a modest but significant STAT-1 phosphorylation in CD4 and CD8 T cells. Thus, IFN signaling may predict clinical response to high dose IFN therapy and should be considered a novel tool for patient monitoring during clinical trials. It is surprising to observe that the analysis of a single pathways (STAT-1) is such a powerful biomarker of immune responsiveness considering the complexity of the JAK/STAT family interactions and their mutual modulation [105,188]. However, it is remarkable that the STAT-1/IRF-1/IL-15 axis is a central component of TSD confirming its relevance to cancer rejection. The general immune suppression of cancer patients had been previously described by other studies, for instance, Heriot et al [189] observed that monocytes from patients with colorectal cancer produce low levels of IFN-α and TNF-α in response to LPS stimulation compared with matched healthy donors. Interestingly, as observed by Lee at al [183], such depression of innate immune responses were observed at early stage in patients with Duke's A and B.

## Basic insights about cancer immune biology

Much can be learned in human immunology by a comparative method that looks at immunological phenomena with an interdisciplinary approach [124]. The relevance of IFN signatures in the context of various diseases represents a good example. He et al [180] observed that decreased IFN signaling and decreased *ex vivo *responsiveness of PBMCs to IFN-α stimulation were harbingers of non-responsiveness of HCV-infected patients to systemic administration of pegylated IFN-α and Ribavarin. These differences were interpreted as related to the genetic background of patients as it was observed that PBMCs from patients of African American (AA) origin were least likely to respond to IFN-α stimulation *ex vivo *and to recover from hepatitis compared to patients of European American (EA) background. This observation raises the question of whether patients with melanoma or HCV that have better changes to respond to therapy are characterized by a different genetic background compared to those likely to do poorly. A recent analysis performed in our laboratories (Pos et al. in preparation) failed to demonstrated dramatic differences between the responses of the two ethnic groups to IFN-α (see later). Thus, alterations in IFN signaling are likely to represent a secondary effect due to the presence of cancer cells or viral particles that in turn may interfere with the innate immune response of the host. This being the case, it will be likely in the future that more insights about the mechanisms leading to altered IFN signaling in cancer patients will be gathered by a more in depth analysis of cancer biology and the products released by cancer cells that may affect immune cells activity locally and at the systemic level.

Indeed tumors, including melanoma, display strong differences in the expression of ISGs [190,191], which are coordinately associated with the expression of several chemokines, cytokines, growth and angiogenic factors [190,192]. Moreover, the presence of immune activation has been associated with the prognosis of melanoma [193]. Thus, it is likely that melanoma and other cancers express an immune modulatory phenotype that may alter not only their own microenvironment but whose effects can reverberate at the systemic level. Whether these differences are due to distinct disease taxonomy [194] or to disease progression [126,190] remains to be clarified.

Mohammed Kashani-Sabet proposed a model that may explain the dichotomy observed in the biological pattern of melanomas. Studying check points in the progression of melanoma, it was observed that BRAF mutations occur early in the development of the disease and do not account for the switch to an increasingly more aggressive phenotype. Transcriptional analysis was performed to compare radial to vertical growth, which identified predominantly loss of gene expression [195,196]. Two subtypes of melanoma were identified that could not be segregated only on account of BRAF mutations. Rather, modifiers associated with the vertical growth phase included immune regulatory genes such as IFI16, CCL2 and 3, CXCL-1, -9 and -10. These genes are up regulated in primary melanoma compared with nevi but become down-regulated in the metastatic phase in some but not all melanomas [195], a phenomenon we had previously observed comparing the transcriptional profile of melanoma metastases to normal melanocytes [190] and other cancers [192]. A multi-marker diagnostic assay for melanoma was developed [197]; a large training set of tissue microarrays with 534 samples including nevi and melanoma biopsies was validated on 4 independent test sets and found ARPC2, FN1, RGS1, SSP1 and WNT2 to be over-expressed in melanoma compared with nevi. Based on the 5 markers, a diagnostic algorithm was developed that could differentiate with high accuracy and specificity benign from malignant lesions [197]. The markers were also evaluated on independent cohorts including the German Cancer Registry (Heidelberg/Kiel cohort). The multi-marker approach tested at several stages of disease could predict sentinel node status and disease specific survival (p < 0.001). The multi-marker score demonstrated higher accuracy than lesion depth or ulceration. A molecular map of melanoma progression is being built from melanocyte to various growth phases and metastatization and will be evaluated in the ECOG data set. Although this algorithm does not directly address the immune responsiveness of tumors, it will be important to include such information for patient stratification in future clinical trials to interpret immunotherapy results.

Constitutive activation of immune regulatory mechanism was also reported by Yutaka Kawakami, who discussed the molecular mechanisms of cancer cell induced immune-suppression and their potential as biomarkers of responsiveness to immunotherapy. In particular, regulatory mechanisms dependent on the MAPK, WNT and BRAF mutations were discussed. BRAF and NRAS mutations occur early in melanoma [198]. Kawakami reported that inhibition of BRAF or STAT-3 depleted the expression of several cytokine including IL-6, CXCL8/IL-8 and IL-10 by cancer cells. Also a MEK inhibitor blocked the expression of IL-10. Finally, VEGF expression was inhibited by small interference RNA (siRNA) for ERK1/2. *In vivo *studies, observed that inhibition of ERK induced the enhancement of T cell responses and protection of mice from cancer [199]. Considering the recently described role of VEGF as a negative predictor of immune responsiveness of melanoma metastases to high dose IL-2 therapy [9] and a poor prognostic marker of survival in colorectal cancer [141], it is possible that this observation may provide an important target for a combination therapy for VEGF expressing melanomas. In particular, the melanoma cell line, 888-MEL previously extensively characterized [200,201] was found to be sensitive to MEK inhibition. Moreover, Kawakami reported that IL-10 production is strictly dependent (in this cell line) upon the expression of β-catenin a mutation inducing enhanced activation of the WNT pathway [202]. Transfection of β-catenin induced production of IL-10; moreover, culture of DC with supernatant of melanoma cells with high β catenin induces IL-10-producing DC and it was decreased by siRNA blockade of β-catenin. Functionally, T cells produced less TNF-α when stimulated with DC cultured with supernatant from β-catenin positive melanomas and expressed higher levels of FOX P3. In a xenogenic model, the human melanoma cells 397-MEL that do not express constitutively high levels of activated β-catenin, were transfected to produce IL-10. Upon antigen exposure T cells were observed to produce less IFN-γ and display lowered lytic activity in animals implanted with the IL-10 expressing tumors. However, IL-10 blocking antibodies did not reverse the tolerogenic effect suggesting that a more complicated mechanism is responsible for the effect on T cells than the direct activity of IL-10. Of interest is the relationship between IL-10 expression and responsiveness. The high expression of IL-10 by 888-MEL contrasts with the observation that this cell line was derived from a patient who dramatically responded to immunotherapy and was a long-term survivor [203]. However, the perceived immune suppressive role of IL-10 may be more complex than previously reported. We observed, that IL-10 expression by melanoma cells studied in pre-treatment biopsies is a positive predictor of tumor responsiveness to immunotherapy with high-dose IL-2 [126,204,205]; moreover, the majority of pre-clinical models in which the effect of IL-10 was evaluated as a modulator of tumor responsiveness identified this cytokine as a factor favoring tumor regression suggesting a dual role of IL-10 promoting growth in natural conditions but favoring tumor rejection upon immune stimulation [206]. Kawakami's work may shed light on this paradoxical observation; screening of siRNA against 800 kinases was done to identify which are involved in immune suppression; it was found that STKX kinase inhibits IL-10 and TGF-β production. Moreover, epithelial-mesenchymal transition is induced by SNAIL transfection, which also induces IL-10, VEGF and TGF-β and, in co-culture with human PBMCs, induces FOX-P3 expression. Co-culture of PBMCs with melanoma cells transfected with SNAIL increases the number of FOX-P3-expressing T cells and this is also reversed by SNAIL/TSP (downstream of SNAIL) blockade. Blocking SNAIL expression by tumors with siRNA induced increase in CD4 and CD8 T cells, thus *in vivo *SNAIL may be involved in immune suppression. Similar results can be obtained by anti-TSP1 which can induce better T cell infiltrates. SNAIL transfected melanoma is resistant to immunotherapy in mouse models and may represent a new predictive biomarker of tumor responsiveness to immune therapy [43].

## Host's genetics vs cancer genetics; the riddle of tumor immunology

The relative contribution of the genetic background of the host, the genetic instability of cancer and the effects of the environment on the natural history of cancer is complex. A good example is nasopharyngeal carcinoma (NPC), which predominantly affects specific geographic areas and ethnicities, in particular the Asian Population [207-210]. NPC etiology is clearly linked to Epstein-Barr virus (EBV) infection [211] and the immune response to the EBV infection appears to bear a strong influence in both the natural history of the disease and response to therapy [207,212-218]. A recent observation linked elevated VEGF secretion by the tumor tissue to outcome; in that study, high VEGF secretion correlated with decreased survival. The reason for the prevalence of NPC in specific ethnic groups remains to be conclusively explained but there is evidence that the genetic background of the host plays an important role in familiar and sporadic cases [209-211,218-230]. However, as for most disease etiologies that are influenced by numerous genes, the genetic determinants of disease prevalence and clinical outcome are still not fully understood [231-238]. In particular, cancer immune responsiveness can be influenced by either the genetic background of the host's or by disease heterogeneity [1,239]. Few lines of evidence suggest that the genetic make up of patients may affect the natural history of cancer or its responsiveness to therapy; a polymorphism of the IFN-γ gene was associated with responsiveness to combination therapy with IL-2 therapy and chemotherapy [240]. Others found that variants of CCR5 are predictors of survival in patients with melanoma receiving immunotherapy [161]. More recently, the responsiveness to IFN-α therapy in melanoma was found to be associated with autoimmune disease which in turn could be related to genetic predisposition [241,242]. Recently, Dudley et al [8] reported that the adoptive transfer of tumor-infiltrating lymphocytes with shorter telomeres was associated with a strongly decreased chance of clinical response; although this effect has been explained by a senescent phenotype of lymphocytes, it is possible that genetic variations in the ability to conserve telomere length could be responsible for differences among patients as previously observed for other instances [243-245].

In a broader sense, the heterogeneous response to IFN-α observed among patients with either cancer [182,183] or HCV [180,246,247] can be plausibly explained by inherited genetic predispositions that determine the responsiveness to this cytokine. It has been proposed that single nucleotide polymorphisms in the IFN pathway are associated with the response to IFN-α therapy of HCV [248]. Moreover, ISG polymorphisms have been associated with other immune pathologies and differences in the prevalence of IRF and STAT gene polymorphisms have been associated with the prevalence of systemic lupus erythematosus in AA [249,250]. Alternatively, racial differences in the responsiveness to a given treatment may come from effects that the disease exerts on the host's immune cells, and from differences to environmental exposures. Thus, AA may be genetically less protected against HCV infection for reasons unrelated to IFN-α activity; yet, the higher viral load or other factors associated with worse disease may, in turn, affect IFN-related pathways [180,246,251,252]. Whether the genetic background determines the responsiveness to IFN-α or whether acquired differences in the disease status are responsible for differences in the disease phenotype among populations, can only be answered by studying normal volunteers not bearing a disease, like cancer or HCV, that are known to affect the immune response [118]. Based on the observation that AA patients with HCV infection are the least likely to respond to IFN-α stimulation, we tested whether immune cells from 48 AA and 48 EA normal volunteers matched for age and sex responded differently to IFN-α. We compared the levels of STAT-1 phosphorylation and global transcriptional profile of T cells between the two ethnic groups. The same subjects were genetically characterized by genome wide single nucleotide polymorphism analysis to determine the racial deviation of the two groups. This is an important task considering the genetic diversity of AA and their potential admixture with other ethnic groups [253] Although there was clear separation among AA and EA at the genomic levels, no clear differences could be identified at the functional level (phospho-assays or transcriptional profiling, Pos et al. manuscript in preparation). Thus, it is likely that differences observed in IFN-α responsiveness among different individuals of distinct genetic background or within the same ethnic group affected by cancer or HCV may be secondary to a difference in the disease itself or a difference in the response of the host to the disease, which may affect secondarily the host's immune response. This observation may help interpret differences in tumor immune biology according to race/ethnicity reported by other groups.

Stefan Ambs (NCI, Bethesda, Maryland, USA) reported a comparison of transcriptional patterns between AA and EA in prostate and breast cancer [254,255]. It is noteworthy that AA have higher death rates from all cancer sites combined than other US populations [256]. Ambs also presented an example for race/ethnic differences in the prevalence of a genetic susceptibility locus from published reports. Several genetic variants at the 8q24 cancer locus are most common among subjects with African ancestry and these differences can explain some of the excess risk of AA to develop prostate cancer. In their study, Ambs and coworkers compared 33 AA and 36 EA macro-dissected tumors by transcriptional analysis. Numerous genes were differently expressed between the two patient groups, but the biggest differences were found to be related to genes involved in the immune response and in particular associated with IFN signaling: IFN-γ, STAT1, CXCL9–11 CCL5 CCL4 CCR7, IL-15 and -16, USG15, Mx1, IRF-1, – 8, -2, OAS2, TAP1 and 2. These genes were over expressed in AA suggesting that in those tumors the cancer cells are in an anti-viral state. Interestingly, the expression of these genes in prostate and breast cancer was associated with resistance to chemotherapy and radiation and in general with a worse prognosis [257] bearing the opposite significance than the expression of similar signatures in colorectal cancer [134,135,141]. Their expression is associated with a poor prognostic connotation in the former and a good one in the latter. An explanation for this discordant and opposite observation is lacking. Similar differences in the tumor microenvironment were observed by Ambs studying breast tumors and comparing tumor stroma and micro-dissected tumor epithelium. Those data were further validated by immunohistochemistry in an extended set of tissues [255]. In tumors from AA, an increased macrophage infiltration was observed, using CD68 as marker, and also a higher micro vessel density, as judged by CD31 expression, when compared with EA tumors

Xifeng Wu (MD Anderson Cancer Center, Houston, Texas, USA) emphasized the need for a systematic evaluation of genetic variants in inflammation-associated pathways as predictors of cancer risk and clinical outcome. The evolution of epidemiologic research from traditional to molecular and even more integrative epidemiology has rapidly changed the paradigm of cancer research. The integration of information at the pathway level is necessary because multiple inherited alterations in gene function can have additive effects as part of a pathway and different pathways can act synergistically or in antagonism. Additional assessment of the predicted or documented functional effects of genetic variants in the biology of disease should also be considered in these models. Wu's hypothesizes that the inflammatory response that plays a role in carcinogenesis is modulated by genetic variability. Fifty-nine SNPs in 36 genes were analyzed. SNPs were selected at promoter UTR or coding region segments according to the literature. Several cytokines were selected and were studied in 1,500 lung cancer cases and 1,700 matched controls. Comprehensive epidemiologic information was obtained and 7 SNPs were found to be relevant. Among them, IL-1α and IL-1β positively correlated with lung cancer prevalence in heavy smokers suggesting that deregulated inflammatory response to tobacco-induced lung damage promotes carcinogenesis [258]. Five SNPs were associated with increased risk of developing bladder cancer including MCP1 and IFNAR2 and two variants of COX2 and IL4r (the COX-2 allele was observed to be associated with reduced mRNA expression) [259]. Interestingly, an IL-6 polymorphism was associated with an increased risk of recurrence after treatment with BCG and with poor survival. In another study of about 400 cases of bladed cancer of whom half experienced recurrence after treatment, Wu and coworkers observed that the genes that were associated with risk of developing bladder cancer were also predictor of response; a survival analysis based on a combination of SNPs including those related to IFN genes could predict with a much higher accuracy risk of recurrence compared to clinical parameters and this observation is now under validation studying a 10,000 SNPs of which 400 belong to the already investigated inflammation-related pathways.

### Predictors of responsiveness

Although the IFN pathways seem to be central to TSD, the large experience gained treating patients with adjuvant melanoma with IFN-α has shown limited success. John Kirkwood (University of Pittsburgh Cancer Center, Pittsburgh, Pennsylvania, USA) summarized the long term experience with this treatment emphasizing the importance of sufficiently large randomized studies to obtain conclusive information about usefulness of therapeutics and related biomarkers [15,242,260]. An extensive meta analysis including all phase II trials suggested that while in various trials different outcome biomarkers are identified these are most likely to fail validation as larger patient cohorts are treated [15]. A recent analysis looking for predictive biomarkers in melanoma and renal cell carcinoma [261] suggested that the *ex vivo *ability of IFN-α to revert STAT-1 phosphorylation signaling defects in melanoma patients may be useful [182,183]. In addition, development of autoimmunity during IFN-α therapy is a clear predictor of a 50-fold reduction in frequency of relapse [241]. Finally, the concentration of various soluble factors in pretreatment sera of patients undergoing IFN-α therapy suggested that the pro-inflammatory cytokines IL-1β, IL-1α, IL-6, TNF-α and chemokines CCL2/MIP-1α and CCL3/MIP-1β are elevated in patients with longer relapse-free survival [262]. Together with VEGF and fibronectin potentially predictive of immune responsiveness to high-dose IL-2 therapy [9], these biomarker represent candidate parameters for validation in future trials. High VEGF, together with high IL-6 levels have also been reported as negative predictor of response to bio-chemotherapy [263,264].

This is advancement from previous analyses in which the majority of putative predictors of IL-2 response were related to post-treatment parameters [265,266]. In renal cell carcinoma an additional biomarker has been described, carbonic anhydrase IX, whose expression in pre-treatment lesions may be associated with higher likelihood of response [267]; interestingly, carbonic anhydrase IX is not expressed by melanomas although they display a similar ranges of responsiveness to IL-2 therapy, suggesting, that this molecule may be a biomarker of a particular phenotype associated with responsive lesions but not the determinant of responsiveness [268]. In any case, further validation, together with a better understanding of the biology of these tumors will hopefully enhance the usefulness of these candidate biomarkers.

It has recently been shown that treatment with anti CTLA-4 antibodies can induce clinical responses in few patients previously vaccinated with irradiated, autologous granulocyte-macrophage colony-stimulating factor (GM-CSF)-secreting cancer cells [269]. However, a large phase III study on hormone refractory prostate cancer-bearing patients treated with the same vaccine (but not anti-CTLA-4 antibody) failed to demonstrate effectiveness leading to early termination of the clinical protocol [270,271].

Masahisa Jinushi (The University of Tokyo, Tokyo, Japan) reported the mechanisms hampering vaccine effectiveness and the potentials for combining anti-CTLA-4 therapy. It was observed that GM-CSF-deficient mice are defective in apoptotic cell phagocytosis and develop autoimmune manifestations including pulmonary alveolar proteinosis, SLE, insulitis and diabetes [272]. GM-CSF transduction restores the production of cytokines that regulate T helper cell differentiation (TGF-β, IL-1b IL-4 IL-12p70 and IL-23p19) in response to apoptotic cells. GM-CSF regulates the phagocytosis of apoptotic cells by antigen presenting cells and modulates the function of the phagocyte receptors milk fat globule EGF 8 (MGF-E8), a protein secreted at high levels by melanomas during the vertical growth phase. MGF-E8 has pleiotropic functions in the tumor microenvironment including promoting cancer cell survival, invasion and immune suppression. While GM-CSF regulates T helper cell differentiation by MFG-E8, TLR stimulation suppresses MFG-E8 production by antigen presenting cells resulting in increased allo-mixed lymphocyte reaction in apoptotic cell loaded macrophages-driven splenocytes proliferation [272]. Blockade of MFG-E8 in tumor cells potentiates GVAX therapeutic immunity in the B16 mouse melanoma model. GVAX/RGE (inhibitor of MFG-E8) vaccines decreases Tregs and decreases tumor specific CD8+ T cell effectors with decrease of FoxP3 and increase in CD69 expressing CD8 T cells [273]. MFG-E8 expression in melanoma patients with advanced stage is high and not detected in non advanced stage melanoma and nevi [274]. Thus, MFG-E8 might be considered a negative regulator of GVAX induced immunity by regulating Treg/Teff balance. It is a prognostic factor and may predict response to GVAX and possibly other types of immunotherapy as recently shown by Aloysius el al [275] with various cancers vaccinated with hTERT peptide-pulsed DCs and by Tatsumi et al. [276] in the context of renal cell carcinoma and melanoma.

## Target Selection

The NCI has shown strong interest in developing a systematic approach to the prioritization of agents to be tested in immunotherapy trials including the type of immune response modifier ()()[277,278] or target cancer antigen [279]. Criteria were developed for the selection of each agent with a non-parametric approach receiving feed back from several investigators; however, the ideal antigen and/or biologic modifier and their combination remain to be defined. An ideal candidate target could be considered a protein expressed consistently by cancer initiating cells. Sato et al. [280] described their efforts in identifying such cells among which they describe sperm mitochondrial cystein rich protein and sex determining region Y box-2 protein as potential candidate targets of immunotherapy. They may be used against breast cancer as their expression correlates with poor prognosis and resistance to chemotherapy. Identification of epitopes is underway for HLA alleles common in the Asian population and this novel target could be considered a potential biomarker for patient selection. Another important target expressed by several tumors and potentially associated with the oncogenic process is NY-ESO, a prototype cancer/testis antigen, which induces strong antibody and T cell responses. Extensive work has been done in Japan on patients with esophageal and other solid cancers [281]. NY-ESO was delivered as cholesterol-bearing hydrophobized pullulan nano-particles that absorb the protein and express it in the antigen presenting cells. Humoral and cellular immune responses were elicited in 9 of 13 treated patients and clinical responses were observed in 4 of 5 evaluable patients. Several examples of antigen spreading were observed and a restricted region of the NY-ESO protein was found to be most immunogenic; it is suggested that, for the future, only this region should used for immunization. This is an example of the relevance of careful immune monitoring related to a specific target antigen that provides insights for the design of future clinical trials.

For gastrointestinal tumors, EpCAM, a tumor associated antigen was proposed as a useful target in gastrointestinal cancers. Use of anti-EpCAM may affect tumor stage and progression. Recently a technique was developed to isolate circulating tumor cells using magnetic beads based on EpCAM expression. Cancer cells were isolated from 130 cancer patients and 40 normal controls. Highly significant differences in extractable cells were observed between cancer and normal patients and between patients with or without metastatic disease. The identification of ≥ 2 circulating cancer cells was associated with tumor stage, survival and pleural or peritoneal dissemination. In esophageal cancer cell lines a proliferation assay was performed showing that introduction of EpCAM increases the expression of cyclins suggesting that EpCAM expression accelerates cell cycle and may be an important novel target for the immunotherapy of gastrointestinal tumors. Indeed, anti-EpCAM antibodies decrease tumor growth in animal models and recent clinical trials have been initiated [282,283]. More recently, antibody-mediated targeting of adenoviral vectors modified to contain a synthetic immunoglobulin g-binding domain in the capsid was described that could be used to target tumor-specific antigens expressed on the surface of cancer cells [284].

Furthermore, attention should be put to the status of methylation or acetylation patterns of various genes that may directly or indirectly affect immune function either by down-modulating the expression of putative tumor antigens, or by interfering with immune-regulatory pathways [285-287].

## Summary

It is becoming increasingly apparent that recurrent themes related to the diagnosis, prognosis and responsiveness to therapy are emerging in the context of cancer immunotherapy. Although relatively unrefined, these concepts appear to be valid as they have been reported in concordance by various groups and several of the observed biomarkers represent conceptually similar pathways involved in tissue rejection or tolerance (Table 1). Although, this is only a beginning, it is encouraging to see that among the thousands of biological permutations that could be considered at the theoretical level, direct human observation is providing a tool to restrict the inquisitive mind of scientists to a much more defined circle of possibilities to be explored in the future.
